# Clinical and Population-Specific Insights Into Inherited Retinal Dystrophies From Whole-Exome Sequencing of the Polish Cohort

**DOI:** 10.1167/iovs.67.11.49

**Published:** 2026-09-28

**Authors:** Katarzyna Ognik, Ewelina Cholewińska, Ewelina Witkowska, Przemysław Sołek, Bartosz Sołowiej, Kamil Jonak, Agata Pietras-Baczewska, Beata Gajda-Deryło, Robert Rejdak, Katarzyna Nowomiejska

**Affiliations:** 1 Department of Biochemistry and Toxicology, Faculty of Animal Sciences and Bioeconomy, University of Life Sciences in Lublin, Poland; 2 Genetics Laboratory of the University Center for Laboratory Medicine, Rare Diseases Unit, University Clinical Center of the Medical University of Warsaw, Warsaw, Poland; 3 Department of Animal Food Technology, Faculty of Food Sciences and Biotechnology, University of Life Sciences in Lublin, Lublin, Poland; 4 Department of Technical Informatics, Lublin University of Technology, Lublin, Poland; 5 Department of General and Pediatric Ophthalmology, Medical University of Lublin, Lublin, Poland

**Keywords:** inherited retinal dystrophies, whole-exome sequencing, molecular diagnosis, polish population, pathogenic variants, rare genetic diseases

## Abstract

**Purpose:**

Inherited retinal dystrophies (IRDs) are a diverse group of genetic disorders that lead to progressive vision loss, with non-syndromic and syndromic retinitis pigmentosa (RP) being one of the most common and genetically heterogeneous forms. This study aimed to explore the genetic landscape of IRDs in the white Polish cohort.

**Methods:**

A total of 203 individuals were recruited, including 159 with RP, 21 with Usher syndrome, 16 with cone-rod dystrophy, four with Bardet-Biedl syndrome, and three with Leber congenital amaurosis. Whole-exome sequencing (WES) was used to identify pathogenic variants associated with IRDs.

**Results:**

Pathogenic variants were detected in 132 participants (65.0%), with a diagnostic yield of 63.7% for RP-related mutations. The most frequent causative variants were located in the *ABCA4* and *USH2A* genes, which accounted for 24% and 16% of the identified variants, respectively. Additionally, 24 novel variants of diverse molecular character were identified, including large deletions and frameshift mutations, reflecting substantial heterogeneity in their molecular nature.

**Conclusions:**

This comprehensive genetic analysis not only supports the molecular diagnosis of IRDs in the Polish population but also highlights the usefulness of WES in determining the genetic causes of hereditary retinal dystrophies and identifying new causative variants. Consequently, a dedicated gene panel will be developed to support molecular diagnosis of RP and related IRDs. The collected clinical, imaging, and genetic data may also be useful for future analyses integrating genotype–phenotype correlations, as well as phenotype–phenotype correlations.

Inherited retinal diseases (IRDs), defined by dysfunction or progressive loss of photoreceptors, are disorders characterized by elevated heterogeneity, at both the clinical and genetic levels. They represent one of the most common groups of ophthalmic diseases with a genetic basis, affecting approximately one in 1500 individuals.^1^^,^^2^ The genetic background of IRDs encompasses more than 280 genes and is characterized by complex inheritance patterns, with autosomal-dominant, autosomal-recessive, X-linked, and mitochondrial modes of inheritance described.^1^^–^^3^ Such a broad genetic range translates into significant phenotypic variability, encompassing the types of cells/tissues affected, disease onset, severity, and progression. The protein products of genes implicated in IRDs perform diverse functions within the retina, from structural components forming key elements of retinal cells to essential elements of the phototransduction cascade and components of the retinoid cycle.^3^^,^^4^

The most common form of IRD is retinitis pigmentosa (RP), with an estimated global prevalence of approximately 1:4000 individuals. RP also called rod cone dystrophy, because it results from the primary loss in rod photoreceptors and is later followed by the secondary loss in cone photoreceptors. RP can be classified into nonsyndromic and syndromic forms. The first includes cases limited to the retina, without involvement of other organs or systems. In contrast, the second encompasses conditions such as Usher and Bardet–Biedl syndromes, which cause additional symptoms unrelated to the visual system.^5^ In the early stages of RP, night blindness typically appears first and is followed by a gradual constriction of peripheral vision, which may ultimately lead to legal blindness. The disease is also frequently accompanied by complications such as cataracts and cystoid macular edema.^6^ RP has a highly variable onset from age five years to adulthood, and patients may retain good central vision for many decades. In contrast, Leber congenital amaurosis (LCA) is congenital and severe, with patients exhibiting nystagmus and early vision loss. Cone-rod dystrophy (CRD) is photoreceptor degeneration characterized by early central vision loss, photophobia, and color vision defects, followed by peripheral vision loss and night blindness. Fundus examination features include characteristic macular pigment changes, temporal optic disc pallor, and arteriolar attenuation. CRDs reflect the opposite to RP sequence of events. CRD is characterized by primary cone involvement, or, sometimes, by concomitant loss of both cones and rods that explains the predominant symptoms of CRD. At end stage, however, RP does not differ from CRD.^7^ There is a complex genetic landscape of IRDs where single mutations can lead to different phenotypes and similar clinical manifestations can arise from mutations in different genes.^8^

RP is a genetically heterogeneous disorder caused by pathogenic variants in more than 90 genes, most of which are associated with photoreceptor function and survival, particularly that of rod cells. The following inheritance patterns are distinguished in RP: autosomal-dominant (15%–25% of cases), autosomal-recessive (the most common form, affecting 50%–60%), X-linked (5%–15% of cases), and sporadic cases (up to 50%).^5^ The pathomechanism of the disease is associated with several processes, including photoreceptor apoptosis, dysfunction of the visual cycle, impairment of intracellular signaling pathways, and metabolic disturbances.^4^^,^^6^

A key aspect in the diagnosis and management of IRD is understanding the genotype–phenotype correlation. Different mutations across various genes result in distinct severity of clinical symptoms, rates of disease progression, and the presence of accompanying features. Genotype-phenotype correlations in RP are clinically important, because different inheritance patterns and gene variants can be associated with substantial differences in age at onset, disease severity, and progression; notably, X-linked RP is associated with one of the most severe retinal phenotypes.^5^ It has also been shown that patients with *PRPF31* mutations exhibit incomplete penetrance and variable expressivity, even for the same molecular variant. Moreover, truncating variants in this gene are often associated with earlier onset and a more severe form of retinal degeneration. Consequently, detailed genetic characterization becomes an essential tool for predicting the clinical course of the disease and for designing targeted therapeutic strategies.^1^^,^^5^^,^^6^^,^^9^^–^^12^ In this context, molecular genetic studies provide a reliable means of confirming diagnosis, defining inheritance patterns, and identifying at-risk individuals.

Next-generation sequencing (NGS) techniques, including gene panels targeting RP/IRD and whole exome (WES) or genome (WGS) sequencing, have revolutionized the molecular diagnosis of IRD, enabling the simultaneous analysis of dozens or hundreds of genes with high sensitivity and at an acceptable cost.^13^^–^^16^ The use of NGS in unselected cohorts of patients with RP has been shown to achieve molecular diagnosis in approximately 50%–70% of patients,^17^ and the use of WES, and especially WGS, increases the likelihood of identifying variants in rare and newly described genes.Genetic panels have limited ability to detect structural changes and variants located in specific regions, such as promoters. Compared to genetic panels, WES has a broader analysis scope, whereas WGS offers the most comprehensive analysis of variations, covering virtually the entire genome.^13^^,^^14^ Both WES and WGS allow for reanalysis of data as new IRD genes are discovered, which, together with increasingly refined bioinformatics tools, contributes to a gradual increase in the proportion of resolved cases over time.^17^ Consequently, the increasing availability of NGS and the rapid development of bioinformatics make detailed genetic characterization an increasingly routine element of RP/IRD diagnostics.^13^

There are only few studies regarding the landscape of IRDs in Poland.^1^^,^^17^^–^^19^ These studies used Sanger sequencing of selected genes,^19^ NGS panels^1^^,^^18^ or WES in small group of patients,^17^ although Poland has about 38 million inhabitants. This study aimed to perform WES in a group of patients with a clinical diagnosis of IRD to identify genes and specific pathogenic variants responsible for the development of this disorders in the Polish population.

## Methods

The study cohort consisted of 203 individuals exhibiting clinical features consistent with an IRD. Participants were recruited at the Department of General and Pediatric Ophthalmology, Medical University of Lublin, between July 2023 and October 2024. All the patients were white Polish citizens. The study was conducted with the approval of the Bioethics Committee (Resolution dated December 15, 2022, No. KE–0254/260/12/2022) and adhered to the principles of the Declaration of Helsinki. All participants provided written informed consent before enrolment. Inclusion criteria were as follows: clinical diagnosis of IRD, non-syndromic or syndromic RP (presence of bone spicules and attenuation of retinal vessels on fundoscopy), concentric constriction of the visual field, or ring scotoma, progressive symptoms with a history of night blindness, and markedly reduced or non-recordable a- and b-wave amplitudes on electroretinography, LCA (early-onset RP with severe vision loss, nystagmus, and extinguished ERG within the first six months of life), or CRD (decreased visual acuity, photophobia, impaired color vision, and central scotoma). Exclusion criteria were as follows: age below 16 years, pregnancy, diseases mimicking RP such as posterior uveitis, previous eye trauma, drug toxicity, and carcinoma-associated retinopathy. These pseudo-RP diseases were excluded on the basis of medical and family history, asymmetry, lack of progression and electroretinography results. All the patients underwent full ophthalmological testing including measurement of the visual acuity, visual field examination, wide-field fundus photography and fundus autofluorescence, optical coherence tomography and electrophysiology examination, as well as medical and family history with pedigree recording.

Blood samples were collected from all patients into EDTA tubes and subsequently frozen at –80°C for pending further analyses. Whole blood was transported to the CeGaT genetics laboratory in Tübingen,Germany, where genetic testing was performed. Analysis was performed by WES on the Illumina NovaSeq6000 platform after enrichment of target regions using the CeGaTExomeXtra v.5 panel. Coding variants, as well as selected known pathogenic non-coding variants (UTRs, intronics, and intergenic variants), were assessed according to the ClinVar, HGMD, and CeGaT internal databases. Analysis included the identification of single-nucleotide variants, small indels, and copy number variants (CNVs), and variant interpretation was performed according to the ACMG/ACGS 2020 guidelines (v. 4.01).^20^

## Results

A total of 203 individuals (102 females and 101 males) who met the clinical inclusion criteria underwent whole-exome sequencing. All included patients were of white Polish ethnicity. The majority of the cohort consisted of unrelated probands, and two families were also included, with two siblings each exhibiting clinical symptoms of RP.

Based on clinical diagnosis, the following phenotypic groups were distinguished among the patients: RP in 159 individuals (78.3% of the study cohort), USH in 21 individuals (10.3%), CRD in 16 individuals (7.9%), BBS in four individuals (2%), and LCA in three individuals (1.5%). Because the initial classification was based on clinical examination, this group also included a small number of patients whose molecular findings later suggested phenotypic overlap with other IRD entities, such as *ABCA4*-associated retinopathy or choroideremia.

The gender distribution and mean age at enrollment within each phenotypic group were as follows: the RP group included 82 males and 77 females (51.57%/48.43%) with a mean age of 44.4 years; the USH group included nine males and 12 females (42.86%/57.14%), mean age 42.2 years; the CRD group included eight individuals of each sex (50%/50%), mean age 38.1 years; the BBS group comprised one male and three females (25%/75%), mean age 38.5 years; and the LCA group included one male and two females (33.33%/66.67%), mean age 17.7 years. The highest mean age at the time of referral was observed in the RP group, whereas the lowest was in patients with Leber congenital amaurosis (Table 1).

**Table 1.** Distribution of Patients Across Phenotypic Groups by Sex and Age

Phenotypic Group	Group Size	Number of Males	Number of Females	Mean Age	SD of Age
RP	159 (78.33%)	82 (51.57%)	77 (48.43%)	44.4	14.1
USH	21 (10.34%)	9 (42.86%)	12 (57.14%)	42.2	15.4
CRD	16 (7.88%)	8 (50.00%)	8 (50.00%)	38.1	19.6
BBS	4 (1.97%)	1 (25.00%)	3 (75.00%)	38.5	20.0
LCA	3 (1.48%)	1 (33.33%)	2 (66.67%)	17.7	10.0
Total	203 (100.00%)	101 (49.75%)	102 (50.25%)	42.4	—

BBS, phenotypic group with a clinical diagnosis of Bardet-Biedl syndrome; CRD, phenotypic group with a clinical diagnosis of cone-rod dystrophy; LCA, phenotypic group with a clinical diagnosis of Leber congenital amaurosis; RP, phenotypic group with a clinical diagnosis of retinitis pigmentosa; USH, phenotypic group with a clinical diagnosis of Usher syndrome.

### Molecular Diagnostics–Diagnostic Yield

A resolved molecular diagnosis was obtained in 132 individuals (65.02%), whereas 34 individuals (16.75%) received an uncertain molecular diagnosis. Among patients with a confirmed molecular diagnosis, autosomal recessive (AR) inheritance predominated (58% of resolved cases), followed by autosomal dominant (AD; 27%) and X-linked (XL; 13%). For the purposes of this study, “resolved molecular diagnosis” referred to cases in which variants were classified as pathogenic/likely pathogenic according to ACMG/ACGS criteria and were considered sufficient to explain the patient's phenotype based on the available clinical and molecular data, specifically two variants for autosomal recessive inheritance, one variant for autosomal dominant inheritance, and one variant for X-linked disease. The uncertain molecular diagnosis group included patients in whom one pathogenic or likely pathogenic variant was identified in a gene consistent with the clinical diagnosis, along with a second variant of uncertain clinical significance (VUS) for autosomal recessive disorders, or a VUS for autosomal dominant or X-linked conditions. In 37 individuals (18.23%), unsolved molecular diagnosis was found that could not provide a molecular explanation for the observed clinical symptoms (Fig. 1a; Tables 2a, 2b).

**Figure 1.**
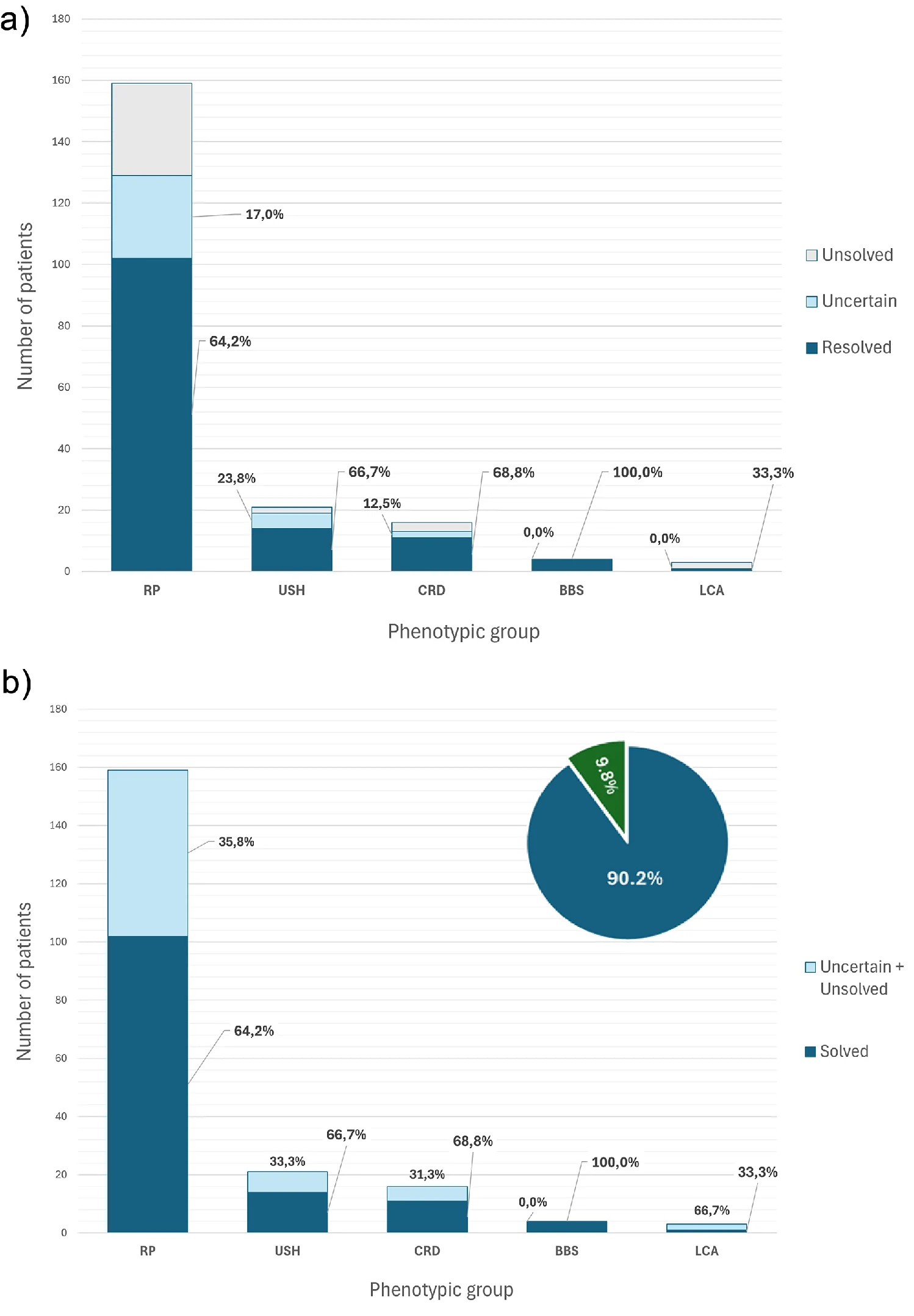
Diagnostic yield and concordance across phenotypic groups. **(a)** Diagnostic yield across major phenotypic groups, including RP, Usher syndrome (USH), CRD, Bardet–Biedl syndrome (BBS), and LCA, showing the proportions of resolved, uncertain, and unsolved cases. **(b)** Diagnostic yield is summarized as resolved versus uncertain/unsolved cases across phenotypic groups, together with overall concordance between clinical and molecular diagnoses in the study cohort. The pie chart shows 90.2% concordant and 9.8% discordant cases.

**Table 2A.** Diagnostic Yield by Phenotypic Groups (Resolved, Uncertain and Unsolved)

Phenotypic Group	Group Size	Resolved	Uncertain	Unsolved
RP	159 (78.3%)	102 (64.2%)	27 (17.0%)	30 (18.9%)
USH	21 (10.3%)	14 (66.7%)	5 (23.8%)	2 (9.5%)
CRD	16 (7.9%)	11 (68.8%)	2 (12.5%)	3 (18.8%)
BBS	4 (2.0%)	4 (100%)	0 (0.0%)	0 (0.0%)
LCA	3 (1.5%)	1 (33.3%)	0 (0.0%)	2 (66.7%)
Total	203 (100.0%)	132 (65.0%)	34 (16.7%)	37 (18.2%)

**Table 2B.** Summary of Molecular Diagnosis Categories in the Studied Cohort

Category	Number of Patients	Importance
Definite molecular diagnosis	132 (65.02%)	A pathogenic variant or variants explaining the phenotype were identified
Diagnosis confirmed	119 (90.2%)	The clinical diagnosis was genetically confirmed
Diagnosis refined/corrected	13 (9.8%)	The clinical diagnosis was not genetically confirmed
Uncertain molecular diagnosis	34 (16.75%)	Variants potentially related to the phenotype were identified, but further validation is required
Unsolved molecular diagnosis	37 (18.23%)	No molecular cause explaining the clinical phenotype was found
Total	203 (100%)	Entire studied cohort

If the group in which a molecular diagnosis is likely but cannot be definitively confirmed in the laboratory without additional data, such as pedigree analysis combined with clinical evaluation of family members, is excluded, 71 patients remain without a molecularly confirmed clinical diagnosis, representing 34.98% of the study cohort. The majority of individuals for whom genetic testing did not yield a positive result were in the group with a clinical diagnosis of non-syndromic RP (57 individuals, including 27 with an uncertain molecular diagnosis, representing 36.3% and 17.2%, respectively). For each phenotypic group, an analysis was also conducted to assess the concordance between the clinical diagnosis based on medical examination and the molecular diagnosis; 90.2% of clinical diagnoses were supported by the genetic analysis. In 9.8% of the study cohort, the resolved molecular diagnosis differed from the clinical diagnosis. The highest concordance was observed in the RP group (95.1%), whereas the lowest concordance was observed in the BBS group, where molecular findings frequently did not fully match the initial clinical diagnosis, reflecting the phenotypic overlap between Bardet-Biedl syndrome and non-syndromic retinal dystrophy. In some patients, variants in BBS genes were associated with retinal dystrophy in the absence of a fully developed syndromic phenotype, which may explain the reduced agreement between clinical and molecular diagnosis (Figs. 1b, 2a; Table 3).

**Figure 2.**
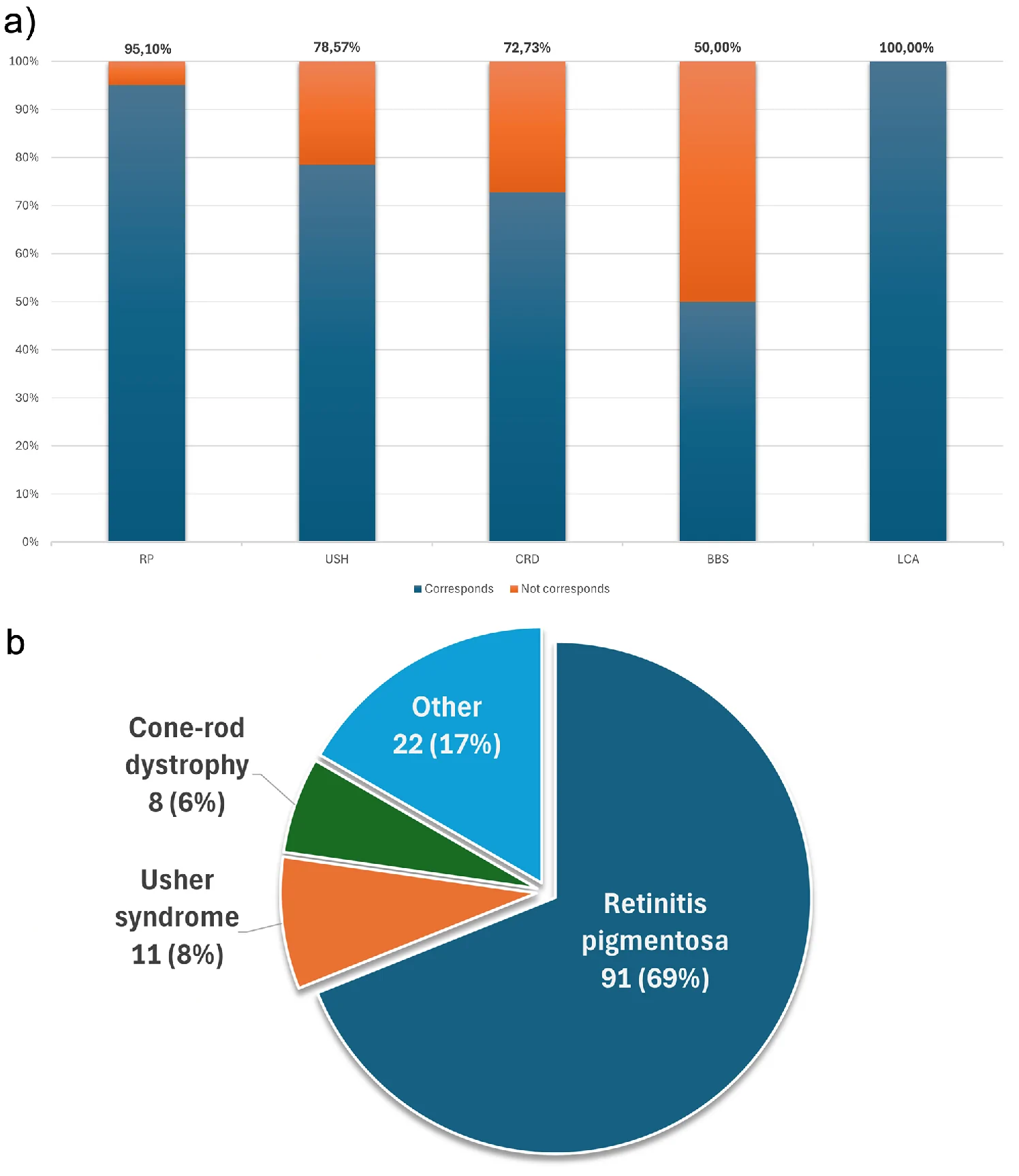
Concordance between molecular and clinical diagnosis in the analyzed cohort. **(a)** Concordance between clinical and molecular diagnoses across phenotypic groups (RP, Usher syndrome [USH], CRD, Bardet–Biedl syndrome [BBS], and LCA) among patients with a confirmed molecular diagnosis. **(b)** Distribution of confirmed molecular diagnoses in the cohort. The number of patients in each subgroup is indicated in parentheses. MD, macular degeneration; SD, Stargardt disease; EL, ectopia lentis; CSNB, congenital stationary night blindness; SDWR, syndromic systemic diseases with retinopathy; CHOR, choroideremia, RPA, retinitis punctata albescens; FA, fundus albipunctatus; NARP, neuropathy, ataxia, and retinitis pigmentosa. The category labeled “RP (digenic)” refers to a predefined subgroup of patients in whom variants in more than one gene may contribute to the phenotype; it does not imply confirmed digenic inheritance, which would require additional familial or functional validation.

**Table 3.** Concordance Between Molecular and Clinical Diagnosis in Phenotypic Patient Groups With a Confirmed Molecular Diagnosis

Phenotypic Group	Group Size	Molecular Diagnosis Is Concordant With the Clinical Diagnosis	Molecular Diagnosis Is Not Concordant With the Clinical Diagnosis
RP	102 (77.27%)	97 (95.10%)	5 (4.90%)
USH	14 (10.61%)	11 (78.57%)	3 (21.43%)
CRD	11 (8.33%)	8 (72.73%)	3 (27.27%)
BBS	4 (3.03%)	2 (50.00%)	2 (50.00%)
LCA	1 (0.76%)	1 (100.00%)	0 (0.00%)
Total	132 (100.00%)	119 (90.2%)	13 (9.8%)

Among the individuals who received molecular confirmation of their observed phenotype, the most frequent genetic changes were those responsible for RP (91 cases [69% of the group with a confirmed molecular diagnosis]), followed by variants causing Usher syndrome (11 individuals [8%]) and cone-rod dystrophy (eight individuals [6%]). In the remaining individuals, the detected molecular variants suggest the possibility of multiple clinical entities or a single clinical entity caused by variants in several genes, which requires further, in-depth clinical reassessment and/or pedigree analysis. There were 22 such individuals in total, representing 17% of the study cohort (Fig. 2b).

### Causative Genes and Variants

Causative variants were identified in 56 genes. They were most frequently found in the *ABCA4* gene, accounting for 24% of all variants. The next most common variants were in *USH2A* (16% of causative variants), *RPGR* (6%), *CDHR1*, *NR2E3* and *EYS* (each 5%), *PROM1* (4%), *RHO* (3%), *PDE6B*, *RP1*, *RP1L1*, *PRPF31* (each approximately 2%), and *BBS5* and *MERTK* (each approximately 1%). Variants in the remaining genes collectively accounted for 22% of causative molecular changes underlying the clinical symptoms observed in the patients (Fig. 3a). The percentage distribution of patients with a molecular diagnosis was as follows: 16.9% of cases carried causative variants in *ABCA4*, 13.4% in *USH2A*, 10.6% in *RPGR*, 5.6% in *RHO*, 4.9% each in *PROM1*, *NR2E3,* and *PRPF31*, 4.2% in *EYS*, 3.5% in *RP*, and 2.1% in *PDE6B*. Variants in *RP1L1*, *CDHR1*, *BBS5*, *MERTK*, *ALPK1*, *PRPF3*, *PRPF8,* and *PRPH2* allowed a molecular diagnosis in a total of 11.3% of individuals in the study cohort. The remaining 25 patients (17.6%) had variants in 25 discrete IRD-associated genes. In the cohort, a subset of patients carried variants in more than one gene potentially responsible for the observed phenotype. However, based on the currently available laboratory findings, it is not possible to definitively determine which variant is causative. This requires additional studies, such as pedigree analysis (Fig. 3b).

**Figure 3.**
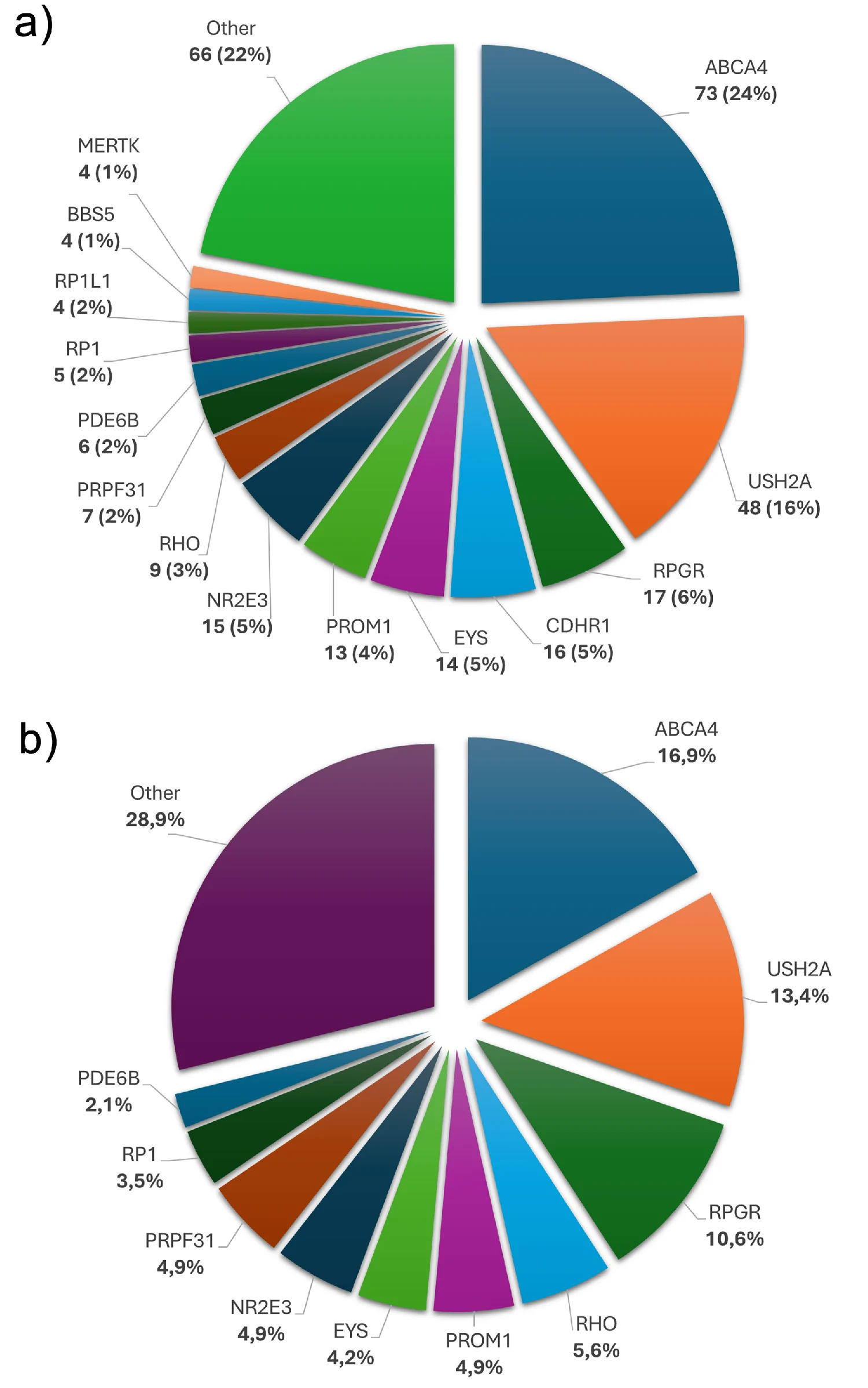
Distribution and classification of causative variants by gene. **(a)** Distribution of causative variants across genes, presented as the proportion of all identified variants attributed to each gene. Other: *CNGA1, IMPG2, AGBL5, ALPK1, BEST1, IFT140, MYOC, PRPF3, PRPF8, PRPH2, ADAMTSL4, ADGRV1, CFAP410, CRB1, FLVCR1, GNAT1, GUCY2D, PCDH15, RDH12, TRNT1, USH1C, USH1G, NRL, AHI1, CEP290, CHM, CLN3, IMPDH1, LCA5, MFRP, MT-ATP6, RBP3, RLBP1, RP2, RPE65, SAG, SNRNP200, TOPORS, PCARE, TULP1, RGR, CNNM4*. **(b)** Distribution of patients with a confirmed molecular diagnosis according to the causative gene. Other: *IMPG2, GUCY2D, NRL, RPE65, BEST1, ADAMTSL4, ADGRV1, CFAP410, CRB1, FLVCR1, PCDH15, RDH12, TRNT1, USH1C, CHM, IMPDH1, MT-ATP6, RLBP1, RP2, SAG, SNRNP200, AGBL5, USH1G, TOPORS, RGR.*

Within our study cohort, we identified a group of patients for whom, based on available evidence, we are currently unable to make a clear molecular diagnosis confirming their clinical symptoms. However, candidate variants were identified in several IRD-associated genes, but these findings should be interpreted cautiously. These variants were most frequently identified in the *USH2A* gene (nine patients [21%]), followed by the *RP1L1* gene (four patients [9%]), and the *RPGR* gene (three patients [7%]). Candidate variants were identified in *RP1L1*; however, given the known complexity of genotype–phenotype correlations for this gene, these findings should be interpreted cautiously and require further clinical and familial confirmation. Other genes in which we identified potentially causative alterations included *PROM1, IMPG2, GUCY2D, ABCA4*, and *PDE6B* (two patients for each gene [5% of the group]). The remaining 17 patients (39%) in this group had candidate variants in 17 different genes (Fig. 4a).

**Figure 4.**
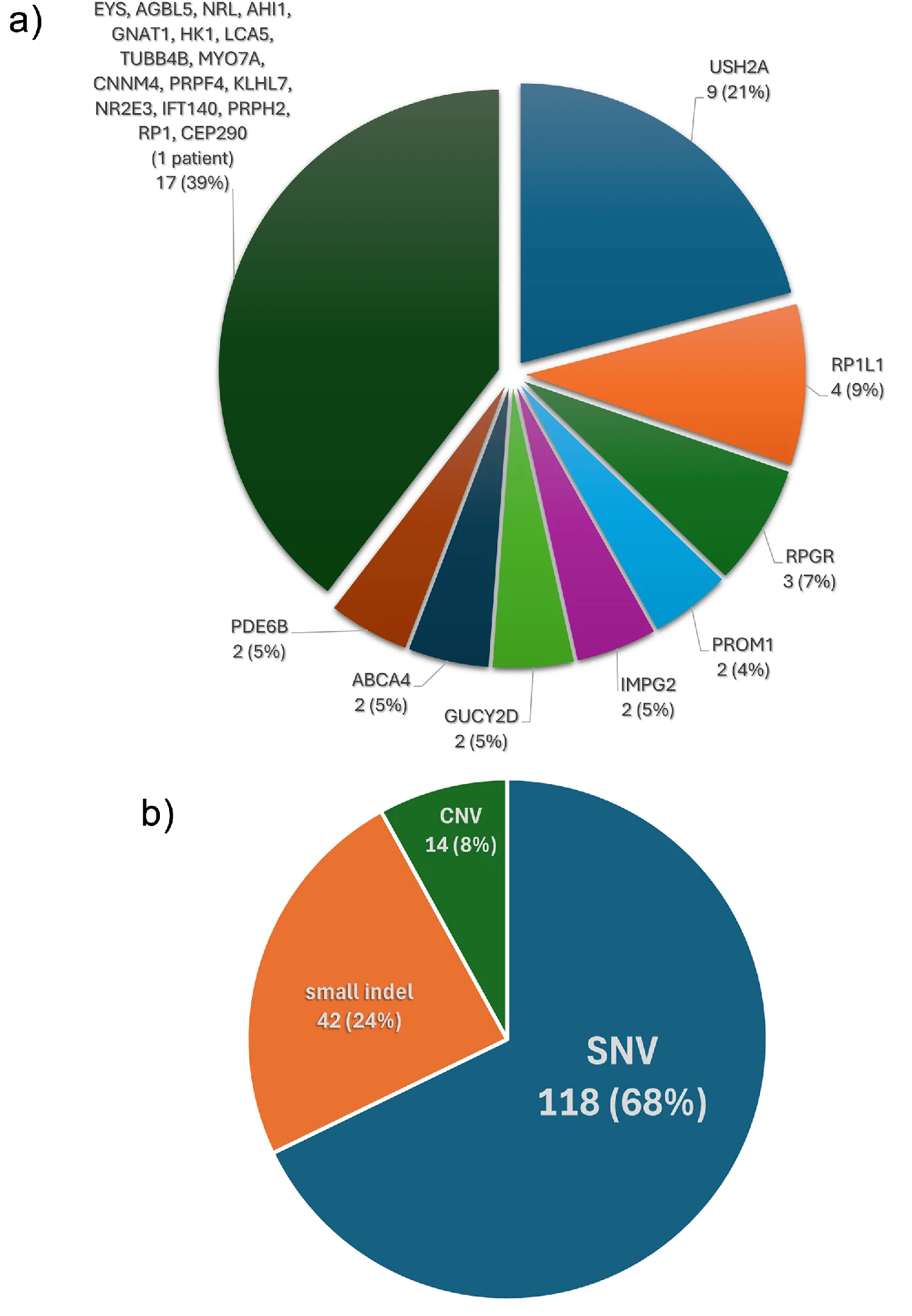
Gene-level distribution of causative and candidate variants. **(a)** Distribution of genes in which causative variants were identified, shown as the number of patients carrying variants in each gene. **(b)** Distribution of variants according to their molecular nature—single-nucleotide variants (SNV), small indel, CNV copy number variants.

Among individuals with a molecular diagnosis, 174 distinct variants were identified. The majority were single-nucleotide variants (single-nucleotide variants, *n* = 118 [68%]), followed by small insertions/deletions (indels, *n* = 24 [14%]) and CNVs (*n* = 14 [8%]) (Fig. 4b).

Considering the potential molecular consequences of the detected variants, the majority were missense variants (*n* = 67 changes [39%]). These were followed by frameshift variants (*n* = 39 [22%]), stop gained variants (*n* = 28 [16%]), large deletions (*n* = 14 [8%]), two in-frame deletions, and one intronic variant located more than five nucleotides from the exon boundary (Fig. 5a). The variants NM_000350.3:c.1622T>C and c.3113C>T in the *ABCA4* gene were the most frequently identified causative changes. In the form of the complex haplotype p.(Leu541Pro;Ala1038Val), they were detected in 13 individuals, two of which were homozygous. Additionally, the c.1622T>C variant was identified independently in one additional patient. The frequency of this complex haplotype (c.1622T>C + c.3113C>T) in the study cohort was 25.8%. Other common *ABCA4* variants included NM_000350.3:c.454C>T (NP_000341.2: p.Arg152*) in four individuals, c.5882G>A (p.Gly1961Glu) in three individuals, and c.5196+1G>A, c.768G>T, c.4919G>A, c.2626C>T and c.1937+1G>A, each observed twice in the cohort. The c.454C>T variant was present as a compound heterozygote in three patients and as a homozygote in one patient. The c.5882G>A variant occurred in one individual as a compound heterozygote with a VUS variant;in contrast, in the other two cases, patients were carriers and had causative variants in other genes. Variants in *ABCA4* observed twice in the cohort were either present as compound heterozygotes or co-occurred with the p.(Leu541Pro;Ala1038Val) haplotype. The remaining variants in the *ABCA4* gene, clinically defined as pathogenic/probably pathogenic, were identified in 16 patients. They occurred in only one allele of the gene and therefore did not provide molecular confirmation of the clinical diagnosis for these individuals.

**Figure 5.**
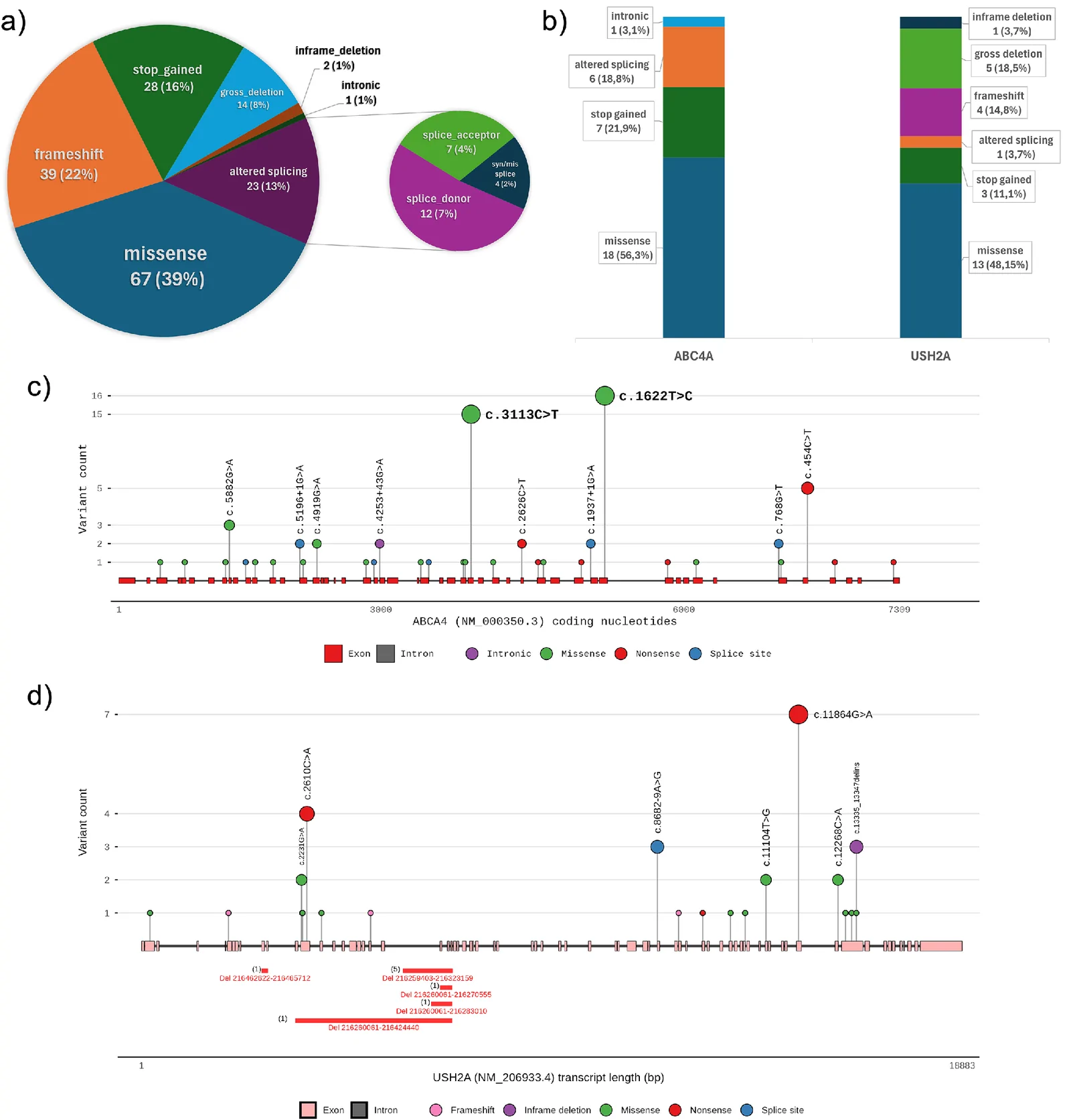
Molecular characteristics and gene-specific distribution of variants. **(a)** Distribution of all causative variants according to predicted molecular consequence. **(b)** Distribution of variant types in *ABCA4* and *USH2A*. **(c)** Distribution of variants along the coding sequence of *ABCA4*. **(d)** Distribution of variants along the coding sequence of *USH2A*.

In terms of potential molecular consequences, the largest group consisted of missense variants (56.3%), followed by stop-gained variants (21.9%), splicing-affecting variants (18.8%), and intronic variants (3.1%). The second most frequently affected gene was *USH2A*, accounting for 13.4% of diagnoses. The most common variant was NM_206933.4:c.11864G>A (NP_996816.3: p.Trp3955), identified seven times; six individuals carried it as compound heterozygotes, and one was a carrier. Other recurrent variants included NM_206933.4:c.13335_13347delinsCTTG (p.Glu4445_Ser4449delinsAspLeu), c.2610C>A (p.Cys870), and c.4627+25435_4987+658del (large deletion), each detected three times. The first variant occurred as a compound heterozygote, the second was present twice with another variant and once as a homozygote, and the large deletion appeared twice in a homozygous state and once as a heterozygote. Interestingly, two other CNV-type variants were reported in this region but differed slightly in breakpoints, likely because of technical limitations of the method used, requiring confirmation by a higher-resolution CNV detection technique. Variants NM_206933.4:c.12268C>A (p.Pro4090Tyr), c.2231G>A (p.Cys744Tyr), c.8682-9A>G, c.11104T>G (p.Trp3702Gly), and c.3157+3_3157+5delinsTAA were each detected twice, all as compound heterozygotes, except for c.8682-9A>G, which was found as a homozygote in one individual. Regarding molecular consequences, the largest group consisted of missense variants (48.15%), followed by large deletions (18.5%), frameshift variants (14.8%), stop gained variants (11.1%), and both splicing-affecting and in-frame deletions (3.7% each) (Figs. 5b–d).

The analysis also identified 24 novel variants that were not recorded in any human genome databases at the time of the study (Table 4). They were most frequently found in *USH2A* (*n* = 5 variants [20.83%]), followed by *PRPF31* (*n* = 4 [16.67%]), *RPGR* and *EYS* (*n* = 3 each [12.5% of this group per gene]), and *ABCA4* (*n* = 2 [8.33%]). The remaining variants were single-patient changes occurring in several other genes (*MERTK, RP1L1, FLVCR1, PCDH15, SAG, USH1C, CNNM4*), each representing 4.17% of the total group. In terms of molecular consequences, these included large deletions (*n* = 9 [37.5%]), frameshift variants (*n* = 8 [33.33%]), missense variants (*n* = 6 [25%]), and one potentially splicing-affecting variant (4.17%) (Fig. 6a). A pathogenic variant in mitochondrial DNA (NC_012920.1: m.8993T>C; YP_003024031.1: p.Leu156Pro) was identified in only one patient (1%). In one patient, two variants were detected in the *RP1* gene: NM_006269.2:c.2029C>T and c.615+3G>A. The first is classified as pathogenic, whereas the second is of uncertain clinical significance (VUS). Pathogenic changes in this gene are responsible for the clinical manifestations of RP and can be inherited either in an autosomal dominant or recessive manner. To precisely determine the clinical significance of the detected variants and the mode of inheritance, further analyses, such as segregation studies in family members combined with detailed clinical evaluation, would be required. Therefore, for this individual, an additional category of possible inheritance, AR/AD, was designated (Fig. 6b).

**Table 4.** Novel Causative Variants (Not Reported in ClinVar and dbSNP)

Gene	Number of Patients With the Variant	HET/HEMI	HOM	Coding Reference Sequence	HGVSc	Protein Reference Sequence	HGVSp	Consequence	Exon/Intron
*USH2A*	3	1	0	NM_206933.4	Deletion of coding exons 21-23	N/A	c.4627+25435_4987+658del	Gross_deletion	21-23
*PRPF31*	3	1	0	NM_015629.4	Deletion of coding exon 4	N/A	N/A	Gross_deletion	4
*USH2A*	2	2	0	NM_206933.4	c.11104T>G	NP_996816.3	p.Trp3702Gly	Missense_variant	57
*MERTK*	2	1	2	NM_006343.3	Deletion of exon 15	N/A	N/A	Gross_deletion	15
*ABCA4*	1	1	0	NM_000350.3	c.3806T>C	NP_000341.2	p.Leu1269Pro	Missense_variant	24
*ABCA4*	1	1	0	NM_000350.3	c.3214G>T	NP_000341.2	p.Val1072Phe	Missense_variant	21
*USH2A*	1	1	0	NM_206933.4	c.4368_4369delTT	NP_996816.3	p.Ser1457Glyfs*19	Frameshift_variant	20
*USH2A*	1	1	0	NM_206933.4	c.9067_9070dup	NP_996816.3	p.Leu3024Hisfs*30	Frameshift_variant	46
*USH2A*	1	1	0	NM_206933.4	Deletion of exons 11-23	N/A	N/A	Gross_deletion	21-23
*RPGR*	1	1	0	NM_001034853.2	c.2597_2598delAA	NP_001030025.1	p.Glu866Glyfs*212	Frameshift_variant	15
*RPGR*	1	1	0	NM_001034853.2	c.1150_1154delTGCTT	NP_001030025.1	p.Cys384Ilefs*9	Frameshift_variant	10
*RPGR*	1	1	0	NM_000328.3	c.434T>C	NP_001030025.1	p.Leu145Pro	Missense_variant	5
*EYS*	1	1	0	NM_001142800.2	c.6291_6297dup	NP_001136272.1	p.Pro2100Cysfs*32	Frameshift_variant	31
*EYS*	1	3	0	NM_001142800.2	Deletion of exons 29-32	N/A	N/A	Gross_deletion	29-32
*EYS*	1	1	0	NM_001142800.2	Deletion of exon 29	N/A	N/A	Gross_deletion	29
*PRPF31*	1	1	0	NM_015629.4	Deletion (1 copy) including exons 1-6 of gene PRPF31	N/A	N/A	Gross_deletion	1-6
*PRPF31*	1	1	0	NM_015629.4	c.1080_1089del	NP_056444.3	p.Lys361Serfs*5	Frameshift_variant	11
*PRPF31*	1	0	1	NM_015629.4	Deletion of exon 8	N/A	N/A	Gross_deletion	8
*RP1L1*	1	2	0	NM_178857.6	c.1056_1082delinsGCCCTCAC	NP_849188.4	p.Ser353Profs*4	Frameshift_variant	4
*FLVCR1*	1	1	0	NM_014053.4	c.733A>T	NP_054772.1	p.Asn245Tyr	Missense_variant	1
*PCDH15*	1	0	1	NM_033056.4	Deletion of coding exon 3	N/A	N/A	Gross_deletion	3
*SAG*	1	1	0	NM_000541.5	c.944+2T>C	N/A	N/A	Splice_donor	11
*USH1C*	1	0	1	NM_153676.4	c.175_178delTTTG	NP_710142.1	p.Phe59Metfs*6	Frameshift_variant	3
*CNNM4*	1	1	0	NM_020184.4	c.725T>G	NP_064569.3	p.Leu242Arg	Missense_variant	1

N/A, not applicable.

**Figure 6.**
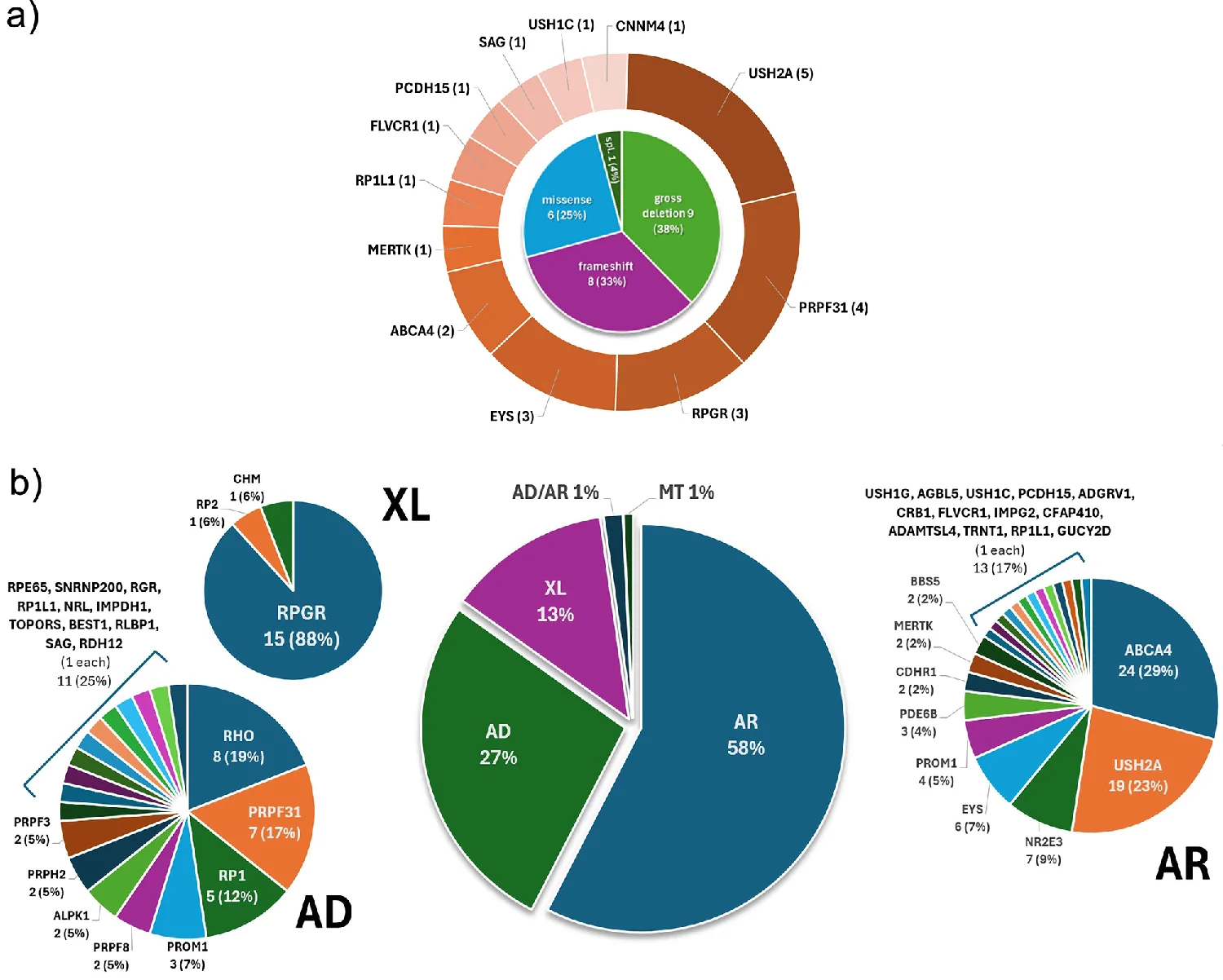
Novel variants and inheritance patterns in confirmed molecular diagnoses. **(a)** Distribution of novel variants across genes, including their predicted molecular consequences. **(b)** Distribution of inheritance patterns among patients with a confirmed molecular diagnosis, including AR, AD, X-linked (XL), and rare mitochondrial or mixed inheritance patterns.

### Interesting Clinical Cases

Molecular analyses conducted in the studied cohort revealed several clinically interesting cases. The first involves a patient in whom two variants in the *AGBL5* gene were identified: NM_021831.6:c.1801C>T and c.626C>T (NP_068603.4:p.Arg601 and p.Thr209Met). Although the patient was clinically diagnosed with Usher syndrome, published *AGBL5*-associated cases more often show an Usher-like phenotype, with retinitis pigmentosa and hearing loss, rather than classic congenital Usher syndrome.^21^^–^^23^

The cohort also included two sisters heterozygous for the NM_025144.4: c.710C>T (NP_079420.3: p.Thr237Met) variant in the *ALPK1* gene, which supports a clinical diagnosis of ROSAH syndrome (OMIM no. 614979). This rare autosomal dominant disorder is characterized by several features, including retinal dystrophy, optic disc edema, splenomegaly, anhidrosis, mild pancytopenia, and migraine-like headaches. In affected individuals, childhood symptoms include visual deterioration associated with optic disc swelling and mild ocular inflammation. Progressive vision loss typically leads to severe impairment by the third decade of life. The identified variant is the most frequently reported pathogenic change in this condition. Importantly, ROSAH syndrome has an autoinflammatory component, and establishing this diagnosis may influence clinical management, including consideration of anti-inflammatory treatment in appropriately selected patients.^24^

Another noteworthy case is a patient in whom two *CNNM4* variants were detected, each in a heterozygous state: NM_020184.4: c.725T>G and c.1393T>A (NP_064569.3: p.Leu242Arg and p.Phe465Ile). At the time of analysis, neither variant had been reported in publicly available human genome variation databases and was therefore classified as a variant of uncertain clinical significance. However, according to some ACMG-based criteria, the first variant may be potentially pathogenic. Mutations in this gene are associated with Jalili syndrome (OMIM no. 217080), a rare inherited disorder characterized by the coexistence of two major conditions: CRD and amelogenesis imperfecta.^25^

Another patient was a man clinically diagnosed with RP, in whom molecular testing revealed the hemizygous variant NM_000390.4: c.525_526delAG (NP_000381.1: p.Glu177Lysfs*6) in the *CHM* gene. Pathogenic variants in this gene cause choroideremia (OMIM no. 303100), a rare X-linked inherited retinal disorder that results in progressive vision loss and ultimately blindness. The disease is characterized by degeneration of the choroidal capillaries, retinal pigment epithelium, and photoreceptors. A hallmark of choroideremia is chorioretinal atrophy in the mid-peripheral fundus with relative preservation of the macula. Affected males experience progressive visual impairment (central vision loss, constricted visual fields, night blindness) beginning early in life, eventually leading to complete choroidal and retinal atrophy.^26^^,^^27^

The cohort also included a group of patients carrying variants of pathogenic or currently uncertain clinical significance in several genes. Of particular interest is a 33-year-old woman in whom the following were identified: a homozygous variant NM_033100.4:c.783G>A in *CDHR1*, a heterozygous variant NM_006445.4:c.6928A>G in *PRPF8* and a heterozygous haplotype NM_000350.3:[c.1622T>C; c.3113C>T] in the *ABCA4* gene. The first variant was identified 13 times in our cohort and represents the most common *CDHR1* variant detected. Considering the inheritance patterns and the pathogenic character of the identified variants, these genotypes may lead to RP (consistent with the patient's clinical diagnosis), as well as cone-rod dystrophy, which correlates with *CDHR1*-associated disease. Therefore a thorough clinical evaluation of the patient, accompanied by pedigree analysis of her family, is strongly recommended.

## Discussion

In our cohort of 203 patients with clinically confirmed retinal dystrophies, WES identified a molecular cause of disease in 65.02% of cases. This diagnostic yield is in line with other IRD studies across different populations, including European cohorts, and supports the value of WES as a robust first-line approach for genetically heterogeneous retinal dystrophies, particularly RP. Consistent with previous reports, pathogenic variants were most frequently detected in genes such as *ABCA4* and *USH2A*.^1^^,^^3^^,^^28^^–^^34^

Our findings regarding *ABCA4* genetic variants are consistent with those reported by Ścieżyńska et al. (2016) and Matczyńska et al.^1^ and also demonstrated high prevalence of the p.(Leu541Pro;Ala1038Val) haplotype in this gene.^35^ The allele frequency for confirmed variants in our cohort was calculated to be 25.8%. Our results are consistent with those for Eastern European populations, but they differ slightly from results for large cohorts selected from other parts of Europe, such as the United Kingdom.^33^ Similarly, it has been shown that the most recurrent *USH2A* variant (the stop-gain c.11864G>A, p.Trp3955Ter) is specific to Eastern and Central European populations.^1^^,^^36^ Our results closely align with previous reports regarding the frequency of large deletions affecting this gene. In total, we identified five deletions spanning exons 21-23, three of which exhibited identical breakpoints detectable using the methodology applied in our study. All of these alterations were found in unrelated patients, and in two cases, they were present in a homozygous state.^1^^,^^31^^,^^37^^–^^40^ Although these two homozygous patients were not known to be related, their shared genotype may indicate local enrichment of the variant in this population and warrants further phenotypic and familial investigation. As with the gene *ABCA4*, other variants characterized as common were identified in other populations.^33^

The clinical RP group was defined at enrollment on the basis of ophthalmological examination. As expected in a genetically heterogeneous cohort, some patients later showed molecular findings compatible with phenotypic overlap with other IRDs, including *ABCA4*-associated disease, cone-rod dystrophy, and choroideremia. This does not invalidate the clinical grouping, but rather reflects the known difficulty of distinguishing overlapping retinal dystrophies before molecular confirmation. Our findings are in contrary to results from Western Europe, for instance, the EYS gene appeared to be the most frequently mutated in Portugal^29^ and *USH2A* and *EYS* in Spain.^41^ Our results are consistent with results from the United Kingdom, with ABCA4, USH2A, and GUCY2D being the most frequent.^42^

Based on clinical diagnosis, CRD explained 8% of 203 cases of IRD in the Polish cohort, and it is consistent with other studies. The prevalence of CRDs is estimated at 1/40,000, thus, CRD is about 10 times less frequent than RP.^7^^,^^42^

We further detected novel variants, the most numerous of which were large deletions (*n* = 9), followed by frameshift variants (*n* = 8), missense variants (*n* = 6), and a single variant potentially affecting splicing. Among the first group, a substantial proportion consisted of deletions spanning repetitive regions of the *USH2A* gene, which may correspond to previously reported findings in the Polish population.^1^ The breakpoints of these deletions differed among the identified variants, which may reflect limitations of the applied methodology. To precisely determine their extent and breakpoint positions, these CNVs would require validation by a higher-resolution method, such as aCGH. Because WES-based CNV calling does not provide base-pair resolution, we cannot exclude the possibility that the variants identified in our cohort overlap with previously reported CNVs in the same genomic region^1^; therefore they should be considered recurrent or likely population-enriched rather than definitively novel until further technical validation is performed. Notably, the concordance between our findings and those reported by Matczyńska et al.^1^ may suggest the presence of CNVs enriched in the Polish population in this gene. Their clinical relevance, however, was supported by the genetic findings and phenotype-based interpretation.

We stated conflicts between phenotypic and molecular diagnoses in 9.8% of cases. They are more likely to be subjective errors in clinical judgement because of clinical heterogeneity of IRDs. These errors were more pronounced for less prevalent IRDs, such as CRD and Bardet-Biedl syndrome. Some patients had more than one potentially causative mutation, which may be explained by the extreme genetic and clinical heterogeneity of IRDs. A second pathogenic variant, often in a different gene, may not cause the disease on its own but can severely modify the phenotype, leading to earlier onset or faster progression, which is often termed oligogenic inheritance. IRDs are caused by over 300 different genes. Because many of these genes work within the same ciliary or phototransduction pathways, a patient might possess mutations in more than one gene that contribute to the same malfunctioning pathway.^8^ Interpreting the pathogenicity of individual variants has become increasingly complex. It has already been shown that the population penetrance of IRD-associated genotypes is markedly lower than traditionally assumed. This suggests that genetic or environmental modifiers are required to manifest disease.^43^

In 37 out of 203 individuals analysed, WES did not reveal any pathogenic variants. Several factors may account for the failure to identify molecular causes of the observed clinical phenotypes. First, there are inherent technical limitations of WES: certain genomic regions, including introns, promoter and enhancer regions, and long repetitive sequences, were not captured. Additionally, regions with high homology or multiple genomic copies may exhibit reduced enrichment or inaccurate variant calling. Low-level somatic mosaicism may also go undetected with standard WES, which is optimized for germline variant detection. Detection of large deletions, duplications, or complex structural variants is not guaranteed with high-throughput sequencing, as such changes may fall outside the captured regions, particularly at potential breakpoint sites. Moreover, benign or likely benign variants were not reported in our results in accordance with current knowledge. Variant classification may evolve as additional evidence emerges and understanding of genetic variation advances. In addition, variants of uncertain significance require external revalidation, including segregation analysis in affected families, re-evaluation in updated variant databases, and additional literature review, before they can be considered definitively disease-causing.

Our analysis was based on sequencing the entire coding regions of the human genome. As a result, certain genomic regions were not covered. According to some reports, the use of WGS may enable additional diagnoses in approximately 10% of cases due to its significantly greater capacity to analyze breakpoints of CNVs and balanced structural variants, as well as to more comprehensively sequence variants in non-coding regions.^20^ Furthermore, it is recommended to re-analyze the obtained data at a later time to reassess the clinical classification of the identified variants.

Based on the results obtained, the most frequent pathogenic variants underlying IRD were identified in a cohort representative of the Polish population. Consequently, a dedicated gene panel could be developed as a population-tailored diagnostic tool to support molecular diagnosis of RP and related IRDs. The collected clinical, imaging, and genetic data may also be useful for future analyses integrating genotype and phenotype. In this context, a targeted gene panel may serve as a cost-effective supplementary approach in selected cases, particularly for the recurrent variants identified in our cohort. However, its clinical performance should be validated in independent cohorts and may vary across populations, and thus broader genomic approaches such as WES or WGS will remain necessary in unresolved or atypical cases.
